# 肺微小脑膜上皮样结节——认知进展与争论

**DOI:** 10.3779/j.issn.1009-3419.2023.102.30

**Published:** 2023-08-20

**Authors:** Haochen LI, Jianchao XUE, Pan LI, Yuan XU, Zhibo ZHENG, Shanqing LI, Naixin LIANG

**Affiliations:** ^1^100730 北京，中国医学科学院北京协和医院胸外科; ^1^Department of Thoracic Surgery, Peking Union Medical College Hospital, Chinese Academy of Medical Sciences and Peking Union Medical College, Beijing 100730, China; ^2^100084 北京，清华大学医学院; ^2^School of Medicine, Tsinghua University, Beijing 100084, China; ^3^100730 北京，中国医学科学院北京协和医院病理科; ^3^Department of Pathology, Peking Union Medical College Hospital, Chinese Academy of Medical Sciences and Peking Union Medical College, Beijing 100730, China; ^4^100730 北京，中国医学科学院北京协和医院国际医疗部; ^4^Department of International Medical Services, Peking Union Medical College Hospital, Chinese Academy of Medical Sciences and Peking Union Medical College, Beijing 100730, China

**Keywords:** 肺微小脑膜上皮样结节, 脑膜瘤, 肺肿瘤, 多原发肺癌, Minute pulmonary meningothelial-like nodules, Meningiomas, Lung neoplasms, Multiple primary lung cancer

## Abstract

肺微小脑膜上皮样结节（minute pulmonary meningothelial-like nodules, MPMNs）是一种与脑膜上皮有相似病理学特征的肺部良性小病灶，和恶性肿瘤有相似的影像学表现，可在临床上导致误诊。关于MPMNs的发病机制尚未达成共识，有观点认为MPMNs可能是一种反应性增生，也有观点认为MPMNs与中枢神经系统脑膜瘤有共同的起源和分子机制。了解MPMNs的特征，深入研究其发病机制，有助于提高对该病的认识和诊断水平。本文就MPMNs的临床、病理、影像学特点以及鉴别诊断和发病机制进行综述，并全面分析了其发病机制的研究进展，对进一步探索提出展望。

## Abstract (en)

Minute pulmonary meningothelial-like nodules (MPMNs) are benign small lesions in the lungs, with similar pathological characteristics to the meningeal epithelium. MPMNs have similar imaging manifestations to malignant tumors, which can lead to misdiagnosis in clinical practice. There is no consensus on the pathogenesis of MPMNs, with some suggest that MPMNs derive from reactive proliferation, while others suggest that MPMNs share a common origin and molecular mechanism with meningiomas in the central nervous system. Understanding the characteristics of MPMNs and studying their pathogenesis will help improve the understanding and diagnosis of MPMNs. In this article, we reviewed the clinical, pathological, imaging characteristics, differential diagnosis and pathogenesis of MPMNs. We also analyze the existing research advances regarding the pathogenesis and propose prospects for further research.

肺微小脑膜上皮样结节（minute pulmonary meningothelial-like nodules, MPMNs）是肺实质中的一类良性小病灶，通常在肺部手术标本和尸检病理检查中被发现，因其与脑膜上皮有着相似的组织结构和免疫组化特征而得名。因MPMNs的影像学表现可类似恶性肿瘤，可在临床上导致误诊。1960年，Korn等^[1]^首次将这些病变命名为肺化学感受器瘤（pulmonary chemodectoma），但后续研究^[2,3]^发现这些病变与脑膜上皮细胞非常相似，因此将其重新命名为“脑膜上皮样结节”。近年来的研究^[2,3]^陆续报道了MPMNs的典型特征和一些罕见表现，但目前对于MPMNs的发病机制，尤其是其与颅内脑膜瘤的关系还存在争论。为引起临床和病理医师以及研究人员对MPMNs的关注，现将MPMNs的研究进展综述如下，并讨论关于MPMNs发病机制一些可能的研究方向。

## 1 MPMNs特征

### 1.1 检出率和临床特征

MPMNs一般被认为是较为罕见，单发或多发，生长缓慢的肺部良性结节，在手术或尸检标本中的检出率为0.07%-13.8%，直径0.1-11.0 mm^[1⇓⇓⇓⇓⇓⇓⇓-9]^。根据近年的研究，MPMNs在单一个体中的检出数目和大小，常常取决于取样和检查方法。

Mizutani等^[10]^对1724例肺切除样本仔细取样（对每个肺叶切除标本通过经支气管输注10%福尔马林固定，切块进行宏观观察，并取10-15个切片全部进行组织学检查），在9.4%（92/976）的肺腺癌患者和12.0%（25/208）的非典型腺瘤样增生（atypical adenomatous hyperplasia, AAH）患者中发现了MPMNs^[10]^。同时Mizutani等^[10]^也揭示了较高的多发性MPMNs出现率（45/121），多发性MPMN平均结节数为4.33个，MPMNs的平均大小在单发和多发的患者中分别为（0.86±0.51）mm和（1.06±0.65）mm。类似的，Mukhopadhyay等^[11]^在每个肺叶进行了27个非肿瘤性实质的切片，在13.8%（69/500）患者中发现了MPMNs，大多数MPMNs的尺寸为1-2 mm，41%（28/69）为多发。

与此相反，如果只关注影像学可见的结节及其周边组织，则获得的检出率较低，且主要限于发现较大的MPMNs，也以单发为主。例如Wen等^[5]^从7589例患者中发现了59例MPMNs患者，22%为多发（13/59），其中43个计算机断层扫描（computed tomography, CT）可见结节平均直径为5.3 mm，远大于Mizutani等^[10]^的研究中报道的1 mm左右。

因此，MPMNs在肺部肿瘤患者，尤其是肺腺癌患者中可能有一定的检出率。在充分检出的情况下，肺切除样本中的MPMNs检出率能达到10%以上，可在接近50%的患者中观察到多发性MPMNs。但由于取材组织块数量不多、影像发现困难等造成了其较低的检出率^[12]^。

关于MPMNs患者的临床特征，一般认为，女性和原发性肺腺癌（或AAH）与MPMNs显著相关，MPMNs好发于60-70岁的患者^[1,10,11,13]^，其发生率在不同肺叶间无显著差异^[10]^，但病变倾向位于外周区，或在叶间胸膜上。除肿瘤外，通过早期尸检研究和外科肺活检，MPMNs与血栓栓塞性疾病的关联也已被注意到^[1,2]^。Mizutani等^[10]^报道，单发和多发MPMNs患者临床特征无显著差异，只是多发MPMNs的患者中的每个MPMNs大于单发病例。在临床实践中，我们还发现MPMNs常在多原发肺癌中出现。

### 1.2 影像学特征

MPMNs在影像上和恶性肿瘤有很强的相似性。一般只有一部分MPMNs在CT扫描中可见（43/79^[5]^、4/12^[14]^），这部分可见的MPMNs多表现为随机分布的纯毛玻璃样阴影，也可混合或实性，界限清晰，直径<5 mm^[15,16]^，也有报道MPMNs可呈现弥漫性薄壁空洞^[17,18]^。经过人工智能（artificial intelligence, AI）扫描和放射科医师确认，可认为与癌无明显区别^[19]^。在我们的患者中也同样观察到了MPMNs，这些MPMNs在CT扫描中均表现为临近胸膜的磨玻璃结节，其中1例有中心空泡影，其CT影像学图片如图1所示。

**图1:**
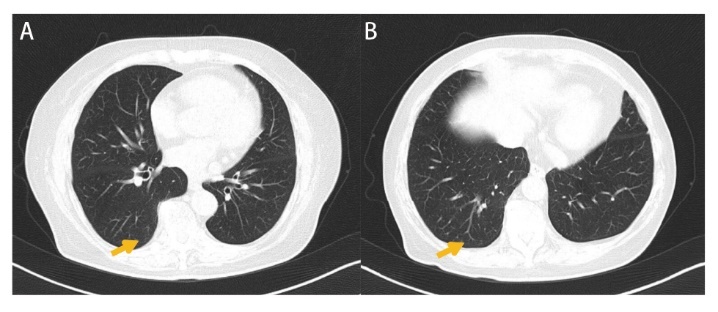
MPMNs的CT影像。患者为右肺下叶多发磨玻璃密度结节，箭头所指为两处结节，病理报告提示为MPMNs，均位于肺周边部，无实性成分与胸膜凹陷征。

18-氟-2-脱氧-D-葡萄糖正电子发射计算机断层扫描（positron emission tomography/CT, PET/CT）可能也无法区分MPMNs和恶性肿瘤。Peng等^[14]^报道有患者术前行PET/CT检查，显示MPMNs最大标准摄取值（standardized uptake value, SUVmax）为4.8，被高度怀疑恶性肿瘤。但我们对带有MPMNs的患者行PET/CT时，未发现MPMNs有代谢增高，可能MPMNs的PET/CT表现还有待进一步研究。

### 1.3 病理学特征

#### 1.3.1 大体特征

MPMNs常没有肉眼可见的异常病变^[4,12]^，有时也可表现为苍白、灰红或褐色的实性结节，硬度中等或偏软，规则至不规则组织，边界有时尚清^[12,19]^。

#### 1.3.2 光镜特征

MPMNs多位于肺周边部，距脏层胸膜较近，在苏木素-伊红（hematoxylin-eosin, HE） 染色下，常可见类似脑膜上皮的细胞，呈巢状或旋涡状排列，间质成分少，高倍镜下常细胞边界不清，中等大小，核圆形或卵圆形，无异型性及核分裂象，有时可见核内包涵体^[4,5,12,20]^（图2A，图2B）。在细胞生长排列方式方面，MPMNs存在沿肺泡间隔生长的特点，这也是和肺内脑膜瘤的鉴别诊断的关键特征之一^[12]^。Wang等^[19]^报道在术中冰冻切片可能不易区分MPMNs，易将其识别为非典型肺泡上皮增生。

**图2:**
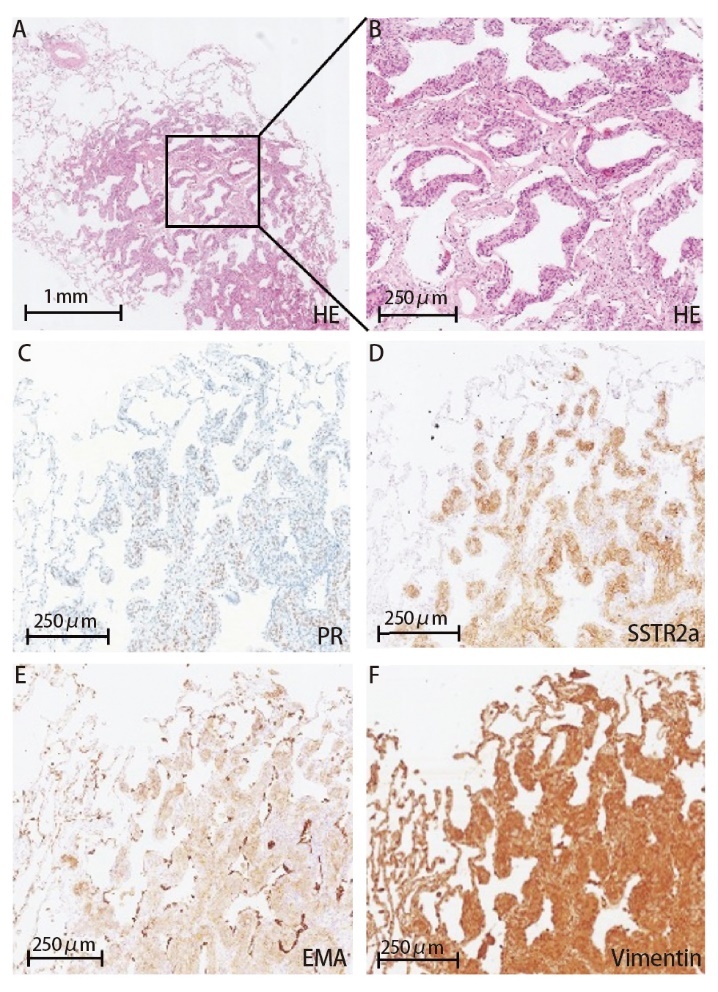
MPMNs组织切片HE染色病理照片和免疫组化照片。A, B：正常肺组织中见一直径1-2 mm的结节，结节中可见上皮样或短梭形细胞巢状排列，中等大小，核圆形或卵圆形，无异型性；C-F：MPMNs免疫组化结果，分别显示PR细胞核阳性，SSTR、EMA、Vimentin细胞质阳性。

#### 1.3.3 免疫组化特征

MPMNs常被发现有类似脑膜瘤的免疫组化表现，上皮细胞膜抗原（epithelial membrane antigen, EMA）、波形蛋白（Vimentin）、孕激素受体（progesterone receptor, PR） 、CD56多呈阳性，而甲状腺转录因子-1（thyriod transcription factor-1, TTF-1）、细胞角蛋白（cytokeratin, CK）、突触素（synapsin, Syn）、中枢神经系统特异性生物蛋白S-100、雄激素受体（androgen receptor, AR）、黑色素瘤相关抗原HMB-45多呈阴性^[5,14,21⇓-23]^（图2B-图2F）。近年来，一些新的脑膜瘤标志物如生长抑素受体2a（somatostatin receptor 2a, SSTR2a）被发现在MPMNs中呈现阳性（图2D），且相比于EMA、Vimentin等其他脑膜瘤标志物有更好的表达率^[22]^，一些新的标志物如胰岛素瘤相关蛋白1（insulinoma-associated protein 1, INSM1），也被证明可用于MPMNs同肺部神经内分泌肿瘤进行鉴别^[23]^。

## 2 鉴别诊断

MPMNs常与原发性肺脑膜瘤（primary pulmonary meningiomas, PPMs）、类癌、不典型类癌等神经内分泌肿瘤、肺血管球瘤、副神经节瘤等相鉴别，其各种特征的比较总结见表1^[5,14,21,22⇓⇓⇓⇓⇓⇓⇓⇓⇓⇓⇓⇓⇓⇓-37]^。

**表1:** MPMNs的鉴别诊断

Differential diagnosis	Image characteristics		Pathological characteristics		Other characteristics
	Size and shape	Number and distribution	Gross and microscopic characteristics	Immuno-histochemical characteristics	
MPMNs	It is a small interstitial lesion that preserves lung architecture, with a diameter usually smaller than 5 mm, growing along the alveolar septa^[24]^	Both solitary and multiple lesions can be found. On CT, solitary lesions are more commonly observed, with a tendency to be located in the peripheral region^[5]^	Often, there are no visible abnormal lesions with the naked eye. Under the microscope, the cells appear to be arranged in a nest-like and swirling pattern, resembling meningeal epithelial cells, with no atypia or mitoses	Positive for EMA, Vimentin, PR, CD56; Negative for TTF-1, CK, Syn, INSM1^[5,14,21,22,23]^	
PPMs	It presents as isolated "coin lesions" with sizes ranging from 0.4 to 6.0 cm. These are large nodular lesions that replace lung parenchyma^[25,26]^	Often solitary lesions	Most of them lack a distinct capsule and appear as well-defined, round nodules with clear boundaries from the surrounding lung tissue. Microscopically, they exhibit typical meningioma-like structures, and sometimes accompanied by psammoma bodies^[25]^	Similar as MPMNs	Very rare^[27]^
Carcinoid	Typically presents on CT as round or oval nodules or masses with clear borders, showing slight lobulation. They can vary in size from 2 to 5 cm and may occasionally exhibit calcification^[28,29]^	They are often central in location and closely related to bronchial anatomy. It can present as intraluminal nodules within the trachea and exhibit the "iceberg sign"	Most commonly, they present as infiltrating or intraluminal masses within the bronchial wall. Generally, the borders are relatively clear or exhibit slight lobulation. There are fewer than 2 mitotic figures, and no necrosis formation^[29,30]^	Neuroendocrine markers (CgA, Syn, CD56, etc.) are positive, and the Ki-67 index is less than 5%	They occasionally present with carcinoid syndrome (commonly characterized by flushing of the face, neck, and upper chest, as well as diarrhea)
Atypical carcinoid	Similar as carcinoid	Similar as carcinoid	The gross characteristics are similar as carcinoid. Microscopically, there are 2-10 mitotic figures observed, or focal necrosis may be present, occasionally appearing as patchy necrosis. However, extensive diffuse necrosis should not be present^[29,30]^	Similar as carcinoid. The Ki-67 index is less than 30%	Similar as carcinoid
Pulmonary glomus tumor	Solid mass with clear borders, rare calcification and cavity formation, ranging in diameter from 1.0 to 9.7 cm. Enhancement shows uneven peripheral enhancement, but lacks central enhancement^[31]^		With clear boundaries, and without a capsule. Compose of numerous small and medium-sized thin-walled blood vessels. Under the microscope, tumor cells are round and regular with clear boundaries. The nuclei are round and located centrally. The stroma may show accompanying hyaline or mucinous degeneration	Positive for SMA, Vimentin and desmin. Negative for neuroendocrine markers^[31]^	
Differential diagnosis	Image characteristics		Pathological characteristics		Other characteristics
	Size and shape	Number and distribution	Gross and microscopic characteristics	Immuno-histochemical characteristics	
Paraganglioma	Generally, a round or oval-shaped mass with relatively homogeneous density, showing mild and uniform enhancement on contrast-enhanced CT^[31,32]^	Also, it can present as multiple nodules in both lungs, often accompanied by local infiltration or lymph node metastasis in the hilum and mediastinum	The lesion has clear boundaries with a capsule. Under the microscope, the tumor cells form a cuff-like arrangement around blood vessels, and rich capillaries in connective tissue can be seen. There are also interstitial septa^[32]^	Positive for S-100, CgA, Syn, NSE^[31]^	
Pulmonary sclerosing pneumocytoma	The shape is regular, density is uniform, borders are smooth, and there is often significant and uniform enhancement^[33]^	It often presents as a solitary nodule or a mass in the peripheral lung area	Translation: The lesion is localized, has a hard texture and are easily detachable. Under the microscope, there are mainly two types of cells: (1) Interstitial round cells (rich cytoplasm, lightly stained, can protrude into the alveolar cavity forming papillary structures); (2) Superficial cuboidal cells (acidophilic cytoplasm, small deeply stained nucleus, covering the surface of papillae and blood vessels)^[34]^	EMA and TTF-1 are positive. In addition, CK7 and Napsin are positive in the superficial cuboidal cells, while Vimentin, PR, and ER are positive in the interstitial round cells	
Type A thymoma	The tumor has a smooth periphery and clear boundaries	Most located in the anterior mediastinum	It often has clear boundaries, intact capsule, and no obvious lobular structure. It is composed of spindle or oval-shaped cells with no significant cellular atypia. It may contain a small number of immature lymphocytes	Positive for CK, CEA, CK19, P63, CK5/6^[35]^	
Metastatic thymoma	It presents as a large mediastinal mass, which can appear as a soft tissue mass with unclear borders	It is mostly located in the mediastinum, accompanied by multiple small nodules in both lungs^[36]^	The tumor lobules are separated by proliferative fibrous septa, forming a leaf-like structure. The lobular structure is not clearly visible in lung metastases^[37]^	Positive for CK, and negative for TTF-1	

PPMs: primary pulmonary meningiomas; TTF-1: thyriod transcription factor-1; CK: cytokeratin; Syn: synapsin; INSM1: insulinoma-associated protein 1; NSE: neuron-specific enolase; ER: estrogen receptor; CEA: carcinoembryonic antigen.

## 3 发病机制

MPMNs的发病机制尚未达成共识。目前主要有两种假说。

### 3.1 MPMNs是一种反应性增生

从临床和病理学特征来看，MPMNs在肺部肿瘤、血栓栓塞性疾病/梗塞等慢性疾病患者中发生率最高，而儿科尸检未发现MPMNs，且MPMNs与小血管无一致性关系，故Mukhopadhyay等^[11]^认为MPMNs可能与潜在的慢性肺部疾病有关。

从基因组学分析来看，Ionescu等^[13]^通过分析杂合性丢失事件（loss of heterozygosity, LOH）比较MPMNs和脑膜瘤的突变损伤情况，发现MPMNs很少出现超过一个LOH，而脑膜瘤表现出高频率的LOH。脑膜瘤的LOH主要位于22q、14q、1p，这些LOH不能在MPMNs中被观测到，提示MPMNs具有反应性起源。另外他们发现多发性LOH仅见于弥漫性肺脑膜瘤病（diffuse pulmonary meningotheliomatosis, DPM），认为DPM可能是反应性增生和肿瘤性增生之间的过渡^[13]^。另外，Niho等^[38]^通过甲基化敏感的限制性内切酶分析了MPMNs的克隆性，11个病灶中5个显示多克隆扩增来源，6个显示单克隆扩增来源，且单克隆和多克隆的MPMNs并无组织学差异。由此他们认为MPMNs可能是反应性增生来源的，而非肿瘤。

### 3.2 MPMNs与中枢神经系统脑膜瘤相关

一方面，MPMNs在病理学特征上与原发性和转移性肺脑膜瘤相似，可被一同归类为胸膜肺脑膜上皮增生（pleuropulmonary meningothelial proliferation, PMP）^[24,39]^。另一方面，在分子层面上，Weissferdt等^[24]^报道MPMNs在常见的遗传途径中与中枢神经系统脑膜瘤相关。脑膜瘤中常见22q神经纤维瘤病（neurofibromatosis type 2, NF2）基因缺失，他们通过荧光原位杂交（fluorescence in situ hybridization, FISH）在部分MPMNs（2/6）和PPMs（1/3）中也发现NF2缺失，提示MPMNs和脑膜瘤有相似的起源和分子途径。

## 4 讨论

MPMNs是一种常在肺部手术标本和尸检病理检查中发现的良性小病灶，大小一般在5 mm以下^[1⇓⇓⇓⇓⇓⇓⇓-9]^。在临床工作中较为罕见，而详细的肺切除样本检查可在10%以上的患者样本中检出直径小至不足1 mm的MPMNs^[11]^。这提示为了发现足够小的MPMNs，得出准确的检出率和临床特征，以及获得足量的MPMNs样本进行深入研究，临床医师需要更仔细地在切除标本中进行寻找。

MPMN的发病机制目前尚无定论。现有研究^[11,13,24,38]^认为在MPMNs产生过程中，反应性增生以及脑膜瘤相关分子途径都有参与。一方面，根据尸检和手术肺切除样本，MPMNs好发于肺部肿瘤、血栓栓塞性疾病、梗塞等慢性疾病患者，而在儿童中不可见。这说明MPMNs的产生是一个慢性过程，也应当有环境因素参与。慢性病可能通过拉伸或加强肺泡隔膜，或通过缺氧、缺血、实质破坏，以及这些因素的某些组合来刺激MPMNs的形成^[11]^。另一方面，MPMNs形成和进展过程中也应当与脑膜瘤有相近的分子途径。MPMNs有类似于脑膜瘤的组织结构和免疫组化特征，也被发现有脑膜瘤相关变异和突变损伤存在^[13,24]^，而且，相较于突变损伤较少的单发MPMNs，DPM有更多的突变损伤^[13]^。基于以上认识，我们将MPMNs发病机制中各层次的已有进展和研究方向总结见表2。

**表2:** MPMNs发病机制进展和研究方向

Research direction	Progress made	Progress direction
DNA	MPMNs have minimal DNA damage but may have deletions in key genes (rarely more than one LOH has been observed, but common NF2 gene deletions have been found)	The mutation profile and methylation status of MPMNs across the entire genome still require systematic research to further identify the key mutations that occur in MPMNs
Environmental factors	Based on the prevalence in certain populations, it is believed that local environmental factors such as ischemia, hypoxia, and mechanical stretching may be involved	Further research is needed to determine the specific environmental factors and their underlying mechanisms
Gene expression	Immunohistochemical staining shows that MPMNs resemble meningiomas rather than lung tissue	Currently, there is a lack of transcriptomic and proteomic data for MPMNs, and further research is needed to investigate the molecular mechanisms underlying the gene expression patterns that resemble meningiomas in MPMNs
Signaling pathways	Based on the NF2 deletions in MPMNs, studies have suggested that MPMNs may share similar growth signaling pathway mechanisms with meningiomas	Further comparisons are needed to explore the similarities in signaling pathway mechanisms between MPMNs and meningiomas
Cellular level	MPMNs are believed to not originate from lung epithelial cells (TTF-1 negative). The primitive cells may be multipotent mesenchymal cells in the subpleural stroma or ectopic embryonic rest cells derived from the dispersed arachnoid cells during embryonic development^[24]^	Further confirmation is needed to determine the cell type serving as the source of MPMNs
Tissue level	MPMNs have a similar tissue structure to meningiomas and are believed to potentially have a growth pattern similar to meningiomas	

LOH: loss of heterozygosity; NF2: neurofibromatosis type 2.

明确MPMNs的形成机制尚需进一步深入研究。第一，MPMNs来源于肺内何种细胞尚不清楚。目前对这一问题的初步认识主要来自免疫组化，例如，正常肺部组织中TTF-1主要在支气管和肺泡上皮细胞表达^[40]^，MPMNs呈现TTF-1阴性说明MPMNs可能不起源于支气管和肺泡上皮细胞，或者在去分化的过程中丢失了TTF-1的表达。真正揭示其来源尚需更加深入的专门研究。解析MPMNs甲基化等表观遗传学景观将会对揭示MPMNs的细胞来源有很重要的意义。此外，近年的研究^[41]^已经逐渐描绘出了人类正常肺的单细胞图谱，利用单细胞图谱结合MPMNs的特征深入搜寻，可能有助于发现MPMNs的原始细胞，并进一步理解这些原始细胞转变为MPMNs的过程。第二，MPMNs产生过程中的关键突变及其积累方式也需要深入研究。目前一些基因组学研究为这一问题提供了初步认识，例如Weissferdt等^[24]^发现的NF2可能是一个关键突变。不过，更多关键突变还需利用先进组学技术进行筛选，以进一步明确MPMNs的发生机制。而且我们还并不清楚这些突变是在MPMNs发生过程中新出现的体细胞突变还是患者本身就有的胚系突变。进一步，我们也不清楚是否有部分人群更容易出现MPMNs以及DPM和PPMs。找到这部分人群的遗传特征有助于更好地做出临床判断。第三，PPMs和MPMNs的关系还需要进一步研究。通过对临床和病理特征的比较以及一些突变分析，目前的研究已经基本认可PPMs和MPMNs之间的密切关系。PPMs的低检出率或许可以通过MPMNs进一步增生需要更多分子和环境条件来解释，PPMs较少与MPMNs共同出现或许是因为较大的PPMs对周围MPMNs存在某些抑制效果。深入分析MPMNs的发病机制以及收集研究更多PPMs样本，能进一步揭示PPMs是否是来源于MPMNs。

了解MPMNs的特征，并阐明其发病机制，有助于深入理解肺对肿瘤等慢性疾病的响应机制，也有助于改善临床应用。第一，MPMNs的影像诊断率还需提高。单纯的MPMNs常不表现出症状，因其与恶性肿瘤在影像学上可有很高的相似性，有时会被误认为恶性肿瘤而进行不必要的手术切除。临床医师应考虑到此类病变，并通过动态监测或侵入性检查区分^[14]^。我们还可以通过提取MPMNs的影像学特征，或从发病机制出发进行影像基因组学研究，以进一步提升MPMNs的影像诊断能力。第二，MPMNs的病理诊断率还有待提高。将MPMNs误认为其他相似病变将大大影响后续治疗。增加对MPMNs特征的了解以及根据发病机制开发具有更好敏感性和特异性的免疫组化标志物，有助于帮助更多病理科医师在进行病理诊断时准确鉴别出MPMNs。第三，DPM患者偶尔会出现限制性肺病综合征^[42⇓⇓-45]^，有时PPMs也可能表现为更具侵袭性的非典型脑膜瘤，可存在胸膜播散^[24]^。对MPMNs发病机制的研究可为相关疾病的治疗提供相应的潜在靶点。

**Competing interests**

The authors declare that they have no competing interests.
